# High usage of telephone telemedicine among people living with HIV at two federally qualified health centers in Los Angeles

**DOI:** 10.1186/s12913-026-14707-8

**Published:** 2026-05-14

**Authors:** Risa Hoffman, Daisy Walker, Derrick Butler, Chi-hong Tseng, Steven Shoptaw, Judith S. Currier, Jay Gladstein, Corrina Moucheraud

**Affiliations:** 1 Division of Infectious Diseases, David Geffen School of Medicine at UCLA, 911 Broxton Ave Ste 301D, Los Angeles, CA 90024 USA; 2 To Help Everyone Health and Wellness Centers, Los Angeles, CA USA; 3 Division of General Internal Medicine and Health Services Research, David Geffen School of Medicine at UCLA, Los Angeles, CA USA; 4 Department of Family Medicine, David Geffen School of Medicine at UCLA, Los Angeles, CA USA; 5 APLA Health, Los Angeles, CA USA; 6 School of Global Public Health, New York University, New York, NY USA

**Keywords:** HIV, Telemedicine, Telehealth, Mixed methods, Quantitative, Qualitative

## Abstract

**Background:**

Federally qualified health centers (FQHCs) in the United States began the widespread use of telemedicine for HIV care during the COVID-19 pandemic. During a mature phase of the pandemic, we sought to increase the uptake of telemedicine, characterize uptake by modality (telephone versus video), and explore the association of telemedicine use with viral suppression.

**Methods:**

We implemented a multi-pronged intervention to increase telemedicine (particularly with video) and performed a prospective cohort study to evaluate this intervention. The intervention included: (1) provision of an easy-to-use video visit platform; (2) training on telemedicine best practices; (3) computer-based and support staff reminders to offer a choice of telephone or video telemedicine; (4) a telemedicine navigator; and (5) quarterly quality improvement meetings. We conducted baseline and endline surveys and chart reviews with a cohort of people living with HIV (PLHIV) to measure telemedicine use (including offer, uptake, and visit completion by modality) and changes in viral load. We used mixed effects regression models to evaluate the intervention’s association with viral suppression over time (< 40 copies/mL).

**Results:**

We enrolled 271 PLHIV between March and November 2022 and implemented the intervention from August 2022-October 2023. Telemedicine visits (telephone and video) in this cohort increased during the intervention period from 13% to 17% of all visits, with this increase predominantly driven by telephone visits. Clinicians’ offer of telemedicine visits (based on participant report) varied, with 36% of clients offered both telephone and video visits during the intervention period, 36% offered telephone visits only, and 28% not offered any telemedicine. Visit completion was highest for telephone visits (82%) and lower for video (58%) and in-person (57%) visits. Viral suppression rates were not significantly different from baseline to endline, regardless of telemedicine use: 71.0% to 77.5% in non-users (*p* = 0.26) and 83.8% to 81.0% (*p* = 0.19) in users.

**Conclusions:**

Our intervention increased the use of telemedicine, predominantly delivered as telephone visits. Additional research is needed on the impact of telemedicine on care engagement over time, on viral suppression over longer follow-up, and on strategies to increase utilization of video visits for PLHIV cared for in FQHC settings.

**Supplementary Information:**

The online version contains supplementary material available at 10.1186/s12913-026-14707-8.

## Background

The COVID-19 pandemic galvanized the use of telemedicine for HIV care at federally qualified health centers (FQHCs) in the United States [1]. The stay-at-home orders of early 2020, paired with the Centers for Medicare & Medicaid Services’ compensation to clinics for telephone and video visits, resulted in many people living with HIV (PLHIV) being offered and receiving HIV care telemedicine services for the first time [2, 3]. Prior to early 2020, telemedicine had not been studied as widely for HIV care as it had been for other chronic conditions, including for mental health, diabetes, and cardiovascular disease – with studies demonstrating equal or better clinical and cost outcomes compared to in-person care for these conditions [4–9]. Research from earlier phases of the COVID-19 pandemic found that FQHCs utilized more telephone visits than video visits compared to clinics serving privately insured patients, but demonstrated that telephone was serving an important role in reaching people that might otherwise be left behind [10]. However, concerns have been raised that telephone telemedicine offers lower quality care than video and may widen health disparities [3, 11, 12].

Our group previously reported that PLHIV receiving HIV care in two FQHCs in Los Angeles during the early phases of COVID-19 were highly satisfied with telephone telemedicine, and many clients were interested in video telemedicine, which was not widely offered [13]. In this same study, clinicians felt telephone telemedicine was beneficial for improving health outcomes for their HIV clients and desired to continue to offer this service – but also were interested in offering more video telemedicine, while feeling that additional support would be necessary to overcome barriers due to difficult-to-use video platforms, complicated workflows for video use, and a lack of sufficient training for both clinic staff and patients [13].

With this background, we designed an intervention to increase telemedicine for PLHIV at two FQHCs in Los Angeles County. Our goal was to evaluate the intervention’s impact on the offer and uptake of telemedicine by modality (telephone versus video) and to understand the role of telemedicine (both telephone and video) on viral suppression. We also sought to explore how sociodemographic factors may be associated with the use of telemedicine overall and by modality (telephone versus in-person).

## Methods

### Definitions

In this study, we defined “telephone telemedicine” as a health care visit between a patient and clinician occurring via phone call, with no video utilized (audio only). We defined “video telemedicine” as a health care visit occurring via video call, where a patient and clinician are able to see each other. “Telemedicine” is used to describe any virtual visit, encompassing both telephone and video telemedicine.

### Setting

The two FQHCs included in the study are located in the Service Planning Area (SPA) 6 of Los Angeles County (south Los Angeles). This SPA has the second-highest rate of new HIV infections in the county [14], and the two FQHCs included in this study serve a population that faces significant barriers to care, including widespread unemployment, lack of stable housing, and prevalent substance use disorders and mental health conditions [13]. In both FQHCs, the same clinical provider delivers care for HIV and other comorbidities (hypertension, diabetes, etc.). At the time the study was performed, most PLHIV at these FQHCs had routine HIV care visits quarterly and both clinics required a minimum of two visits per year (every six months) to receive antiretroviral therapy (ART) refills. With the beginning of the COVID-19 pandemic and stay-at-home orders that were issued in March 2020, both FQHCs began offering telemedicine, predominantly as telephone visits.

### Intervention

We implemented a multi-pronged intervention to improve the offer and uptake of telemedicine, and especially video telemedicine due to both clients’ and providers’ stated interest in this platform [13, 15], consisting of the following: (1) provision of an easy-to-use video visit platform called Doximity (which can also be used for audio only communication) [16]; (2) training for clinicians and clinical support staff on Doximity and telemedicine best practices; (3) computer-based and support staff reminders to offer telemedicine to PLHIV, including offering a choice of telephone or video; (4) an on-site telemedicine navigator to assist clinic staff and PLHIV with telemedicine support, particularly for use of video; and (5) quarterly quality improvement team meetings, i.e., clinicians and support staff at both FQHCs meeting together by Zoom to discuss telemedicine challenges and solutions.

### Study design and outcomes

We performed a prospective cohort study to evaluate the intervention. Individuals were enrolled at baseline and completed a survey about prior telemedicine use, barriers to telemedicine use (telephone and video), and experiences with telemedicine (if any) [15]. Their medical charts were also reviewed for HIV clinical data and utilization of telemedicine HIV care in the prior 12 months (telephone versus video versus in-person). After 15 months of the intervention (approximately 12 months of exposure to the intervention for each enrolled person), participants completed an endline survey (Supplementary File 1) and all charts (including for individuals who could not be located for an endline survey) were reviewed for visit types and dates, and HIV clinical data.

The primary study outcome was uptake of telemedicine by type of visit (telephone versus video). Secondary outcomes were visit completion rates, defined as the proportion of visits completed on the scheduled date by each modality (in-person, telephone, video), and viral suppression rates in telemedicine users versus non-users. Secondary implementation outcomes were acceptability from the client perspective (measured via surveys) and acceptability and feasibility from the clinical provider and managerial staff perspective (assessed via in-depth interviews, Supplementary File 2).

### Study population

PLHIV were eligible for the study if they were at least 18 years old and engaged in HIV care at one of the two study sites, defined as having at least one visit (in-person or telemedicine) in the year prior to enrollment (not including the day of enrollment). Potentially eligible PLHIV were referred to the study by clinicians or case managers at each FQHC.

Clinicians and staff were eligible if they were at least 18 years old; currently involved in the delivery of HIV care at either FQHC as a clinician (prescribing antiretroviral therapy [ART]), case manager, or clinic leader (management role); and had been involved in HIV care at the facility since at least July 1, 2022 (to have had exposure to the intervention). Individuals were identified by the lead site investigator at each FQHC and if interested in participating, referred to the study coordinator for more information about the study and screening.

### Conceptual frameworks and theoretical models

The RE-AIM framework was used to plan and evaluate our intervention with a focus on reach, adoption, and implementation outcomes [17]. PLHIV baseline and endline surveys were also developed using two behavioral theories: Venkatesh et al., which assesses what drives acceptance in groups that might not be motivated to use new interventions [18], and the Theory of Planned Behavior/Theory of Reasoned Action [19, 20] to explore factors hypothesized to be associated with use of telemedicine. We used the RE-AIM framework in combination with classic behavioral theory to guide both the design and evaluation of this telemedicine implementation effort. Classic behavioral theory informed the intervention’s mechanisms of action by identifying key determinants of telemedicine uptake at the individual level – including attitudes toward telemedicine, perceived norms, self-efficacy, and perceived usefulness. RE-AIM provided a complementary framework to assess real-world impact by evaluating not only effectiveness, but also reach to different types of clients, adoption by clinicians, feasibility of implementation, and maintenance over time. Together, this combined approach was utilized to link theory-driven behavior change with pragmatic implementation outcomes, enhancing the intervention’s scalability, generalizability, and relevance for health system decision-making.

Our endline in-depth interview guide was based on the Consolidated Framework for Implementation Research (CFIR) [21], with one guide tailored for clinicians and another for managerial staff (case managers, clinic leadership). Guides included questions related to the following CFIR domains: innovation (what was your experience with telemedicine for HIV care like over the last year in terms of complexity, adaptability, relative advantages, and quality?); outer setting (how do patient needs and resources make someone a good/less good candidate for completing a scheduled video/telephone visit?); inner setting (what is your/the clinic’s readiness for implementation of telemedicine for HIV care [adequate training/resources] and how has the clinic culture helped/hindered implementation?); characteristics of individuals (how has the intervention changed your knowledge and beliefs about telemedicine?); and process (what refinements/adaptations/leadership changes can help support the delivery of telemedicine visits?).

### Data collection and analysis

#### Surveys

Baseline and endline surveys were interviewer-administered, with each question read aloud and answers marked in a tablet using SurveyCTO data collection software [22]. Surveys assessed the following domains: sociodemographic information, including housing status (with stable housing defined as either owning, renting, or sharing but not paying for housing); telemedicine utilization for care (HIV and other care); access to technological resources for telemedicine; and costs associated with receiving HIV care in-person and via telemedicine visits. At endline, questions were focused on experiences with telemedicine care including access to privacy and overall satisfaction with care (based on their rating of the statement “I feel satisfied with the quality of care I’ve received on my HIV telephone/video/in-person visits in the last year” on a Likert scale from 1 to 5, where 1 was strongly disagree and 5 was strongly agree). We assessed technological literacy with smartphones, tablets, and computers using the Telehealth Literacy Screening Tool (TLST) scale [23], where a score of 0 designates the lowest literacy and a score of 16 designates the highest.

Surveys lasted approximately one hour and, depending on participant preference, were performed in either English or Spanish and completed either over the telephone or in-person. PLHIV received compensation for completion of the baseline and endline surveys, but not for any clinical visits during the study follow-up period. In-depth interview participants received a gift card as compensation for time.

#### Chart reviews

At baseline, charts were reviewed for number of chronic medications for non-communicable diseases (e.g., hypertension, diabetes, mental health conditions), number of non-communicable disease comorbidities, most recent CD4 cell count within the last year, and most recent viral load within the last year. After each participant completed one year of follow-up, charts were re-reviewed for all clinic visit dates (excluding emergency room follow-up, urgent care, preoperative, and walk-in visits), modality for each visit (in-person, telephone, video) and completion status (completed, rescheduled, or missed), as well as for all viral load results. Data were collected with Survey CTO and analyzed in STATA, version 18.0 [24].

#### Statistical methods

Summary statistics were generated for baseline survey responses, including sociodemographic and clinical data. Baseline CD4 count was calculated as a median value, and viral load at baseline was reported defining undetectable as < 40 copies/mL. A median technological literacy score was calculated. Summary statistics were also used to describe endline telemedicine experiences, including challenges with privacy and overall satisfaction levels.

The chi-square test was used to compare the proportion of visits completed by modality (in-person, telephone, video) from the pre-intervention period to the intervention period. To assess the association between categorical sociodemographic variables and the completion rate for in-person and telemedicine visits, we first categorized individuals as a user of telemedicine (any telemedicine visit during the intervention period) or non-user (no telemedicine visits). This analysis excluded any individual with baseline data for whom no follow-up visits were performed. For this analysis we combined video and telephone telemedicine into one category (telemedicine visit modality) due to the small number of video visits performed during follow-up. We then used chi-square and t-tests to compare sociodemographics by telemedicine use status.

For assessing viral load outcomes, as participants had multiple viral load measures during the study period, a mixed effects regression analysis was used to evaluate the difference in viral load suppression by telemedicine use, with a random intercept for participants. We evaluated viral load at baseline (defined as within six months prior to the intervention onset) and over the following four quarters. The primary analysis was performed with viral suppression defined as < 40 copies/mL, with a sensitivity analysis using a threshold of < 200 copies/mL. Participants without any baseline or follow-up viral loads were excluded from this analysis.

#### In-depth interviews

Endline in-depth interviews with clinicians and managerial staff were conducted via Zoom and lasted between 30 and 70 min. Interviews were audio-recorded, transcribed, and analyzed utilizing piloted and reviewed codebooks developed by the study team [13] based on CFIR [13, 21]. We performed a thematic analysis by reviewing all coded extracts and identifying common themes. Quotes were identified by respondent type (clinician, clinic leadership, or case manager) and years of experience in HIV care.

#### Ethical review

The study was approved by the Institutional Review Board at the University of California, Los Angeles (IRB #20-001508). Oral informed consent was obtained from both PLHIV and key informants.

## Results

### Demographics: PLHIV

We enrolled 271 PLHIV and performed baseline surveys between March and November 2022; delivered the intervention between August 2022 and October 2023; and completed endline surveys between July and October 2023.

Table 1 summarizes baseline characteristics of the PLHIV enrolled. The median age was 50 years (IQR 38–59), with 79% (*n* = 215) identifying as cisgender men, 17% (*n* = 47) as cisgender women, 3% (*n* = 7) as transgender women and 1% (*n* = 2) as gender diverse. Participants were on ART for a median of 12 years (IQR 7, 21) and most identified as Black/African American (46%, *n* = 126) or Hispanic/Latino/a (26%, *n* = 71), with 86% preferring English (*n* = 232) and 14% preferring Spanish (*n* = 37). A majority of individuals (87%, *n* = 235) reported stable housing, but of those with current stable housing, 34% (*n* = 80) were worried about maintaining this housing over the next three months. Sociodemographic characteristics did not vary significantly by clinic (data not shown).

**Table 1:** Sociodemographic characteristics of study participants (*n* = 271)

	Overall	Clinic 1	Clinic 2
	(*n* = 271)	(*n* = 136)	(*n* = 135)
**Age**, Median (IQR)	50 (38–59)	46 (35–57)	53 (40–62)
**Gender**, n (%)			
Cisgender female	47 (17)	13 (10)	34 (25)
Cisgender male	215 (79)	115 (85)	100 (74)
Transgender female	7 (3)	6 (4)	1 (1)
Gender diverse	2 (1)	2 (2)	0 (0)
**Race/Ethnicity**, n (%)			
Black or African American	126 (46)	57 (42)	69 (51)
Hispanic or Latino/a	71 (26)	25 (18)	46 (34)
Asian	6 (2)	5 (4)	1 (1)
Native American	3 (1)	2 (1)	1 (1)
White/Caucasian	27 (10)	21 (15)	6 (4)
Multi-racial	35 (13)	23 (17)	12 (9)
Other	2 (1)	2 (1)	0 (0)
Prefer not to answer	1 (1)	1 (1)	0 (0)
**Time living with HIV (years)**, Median (IQR)	14 (8–23)	12 (7-21.5)	16 (8–24)
**Time on ART (years)**, Median (IQR)	12 (7–21)	10 (6-19.5)	14 (8–23)
**Preferred language**, n (%)			
English	232 (86)	126 (93)	106 (79)
Spanish	37 (14)	9 (7)	28 (21)
English and Spanish equally	2 (1)	1 (1)	1 (1)
**Sexual orientation**, n (%)			
Heterosexual	96 (35)	33 (24)	63 (46)
Gay	121 (45)	70 (51)	51 (38)
Bisexual	31 (11)	19 (14)	12 (9)
Other (queer, pansexual, asexual)	12 (4)	8 (6)	4 (3)
Prefer not to answer	11 (4)	6 (4)	5 (4)
**Highest level of school completed**, n (%)			
Some school but did not complete high school or GED	55 (20)	19 (14)	36 (27)
High school or GED	149 (55)	78 (57)	71 (52)
College or university	58 (21)	31 (23)	27 (20)
Graduate studies	9 (3)	8 (6)	1 (1)
**Employment status**, n (%)			
Working	104 (38)	56 (41)	48 (36)
Not working - retired	20 (7)	7 (5)	13 (10)
Not working - on disability	70 (26)	36 (27)	34 (25)
Not working - unemployed	76 (28)	37 (27)	39 (29)
Prefer not to answer	1 (1)	0 (0)	1 (1)
**Housing status**, n (%)			
Stable*	235 (87)	116 (85)	119 (88)
Unstable**	35 (13)	19 (14)	16 (12)
Prefer not to answer	1 (1)	1 (1)	0 (0)
**If stable housing**,** worried about maintaining housing in next three months**, n (%)			
Yes, very worried	53 (23)	29 (25)	24 (20)
Somewhat worried	27 (11)	12 (10)	15 (13)
No, not worried at all	156 (66)	76 (65)	80 (67)
**Consistent access to device/network needed to be able to do telemedicine visits**, n (%)			
Able to do video and telephone visits	254 (94)	124 (91)	130 (96)
Only able to do telephone visits	10 (4)	6 (4)	4 (3)
Not able to do either	7 (3)	6 (4)	1 (1)
**Telehealth Literacy Screening Tool (TLST) score**, Median (IQR)***			
Smartphone****	14 (12–15)	15 (13–15)	13 (11–15)
Tablet^^^	13 (12–15)	13 (12–15)	13 (10-14.5)
Computer^&^	14.5 (13–15)	15 (13–15)	14 (12–15)
**Pays out of pocket for transportation to clinic**, N (%)			
Yes	181 (67)	90 (66)	91 (67)
No	90 (33)	46 (34)	44 (33)
**Has taken time off from work for visits**, N (%)			
Yes, paid time off	9 (3)	6 (4)	3 (2)
Yes, unpaid time off	38 (14)	17 (13)	21 (16)
Yes, mix between paid and unpaid time off	6 (2)	3 (2)	3 (2)
No	218 (80)	110 (81)	108 (80)
**CD4 counts in past year**, Median (IQR)			
Cells/mm^3	594 (393–818)	609 (393–863)	584 (391–786)
Percent	30 (23–38)	31 (23–39)	28 (23–37)
**Comorbidities**, Median (IQR)	1 (0–2)	1 (0–2)	1 (0–2)
**Non-ART medications**, Median (IQR)	2 (0–4)	1 (0–3)	3 (1–6)
**Baseline viral load**, n (%)^#^			
< 40 copies/mL	208 (79)	110 (82)	98 (75)
≥ 40 but < 200 copies/mL	25 (9)	10 (7)	15 (12)
≥ 200 copies/mL	31 (12)	14 (10)	17 (13)

*Stable housing is defined as owning, renting, or sharing but not paying for housing

**Unstable housing is defined as staying in transitional housing, a shelter, in a car, or on the street

***Score from minimum of 0 (lowest literacy score) to maximum of 16 (highest literacy score)

****n = 155; only measured for those who use smartphone to access the Internet

^*n* = 32; only measured for those who use tablet to access the Internet

^&^*n* = 43; only measured for those who use computer to access the Internet

^#^*n* = 264 (7 had no viral load lab in year prior to baseline survey)

Of participants who had a viral load in the 12 months prior to the baseline survey (97%, *n* = 264), 79% (*n* = 208) were less than 40 copies/mL, 9% (*n* = 25) were between 40 and 200 copies/mL, and 12% (*n* = 31) were greater than 200 copies/mL. The median baseline CD4 count was 594 cells/mm^3^ (IQR 393, 818).

We conducted endline surveys with 172 of the 271 participants in the cohort (64%). Of the 99 participants who did not complete an endline survey (37%), reasons included: did not respond to survey requests but were retained in care (had at least one visit in the prior year) (*n* = 65, 66%); retained in care but did not agree to be surveyed again (*n* = 2, < 1%); transferred to a different clinic and were not contacted for survey (*n* = 22, 22%); not retained in care (*n* = 4, < 1%); or died during the study period (*n* = 6, < 1%). Overall retention in care for those surveyed at baseline was 98.5%. Demographic characteristics were similar in those who did versus did not complete the endline survey (Supplementary Table 1).

### Telemedicine uptake and completion rates

Over the year prior to the intervention, 60% of participants had at least one telemedicine visit for HIV-related care (162/271) and during the intervention period this remained similar with 54% (138/257 whose charts included a follow-up visit after baseline) participating in at least one telemedicine visit for HIV-related care (telephone or video) (*p* = 0.16). Of those retained in care, 15% of telemedicine users prior to the intervention became non-users during the intervention, and of the 71% of these (29/41) who completed an endline survey, 21% (6/29) were non-users because they were not offered telemedicine. Approximately 22% of pre-intervention telemedicine non-users became users during the intervention.

In the year prior to the intervention, 13% of all visits (251/1903) were done via telemedicine (either telephone or video) and this increased to 17% (309/1810) during the intervention (*p* < 0.001). The increase included both a rise in telephone visits (13% to 16%) and in video visits (< 1% to 1%) from baseline to endline. Visit completion rates decreased for all modalities during the intervention period, but of the three modalities, telephone had the highest completion rate (82%, *n* = 232), followed by video (58%, *n* = 15) and in-person visits (57%, *n* = 850). Table 2 illustrates clinic visit types and completion rates from the baseline versus intervention periods.

**Table 2:** Total visits scheduled by visit type and visit status pre-intervention compared to intervention periods (*n* = 271)

VISITS	Pre-Intervention: March 2021 - July 2022			Intervention: August 2022 - October 2023		
	Overall	Clinic 1	Clinic 2	Overall	Clinic 1	Clinic 2
**Total number of all visits** (***n***)	1903	1046	857	1810	1035	775
**In-person visits (n**,** % of all visits)**	**1652 (87)**	**902 (86)**	**750 (88)**	**1501 (83)**	**849 (82)**	**652 (84)**
*Completed (n*,* % of in-person visits)*	*979 (59)*	*555 (62)*	*424 (57)*	*850 (57)*	*481 (57)*	*369 (57)*
*Rescheduled*	*219 (13)*	*71 (8)*	*148 (20)*	*291 (19)*	*172 (20)*	*119 (18)*
*No-show*	*353 (21)*	*235 (26)*	*118 (16)*	*247 (17)*	*141 (17)*	*106 (16)*
*Other (cancelled or walk out)*	*101 (6)*	*41 (5)*	*60 (8)*	*113 (8)*	*55 (7)*	*58 (9)*
**Telephone visits (n**,** % of all visits)**	**243 (13)**	**144 (14)**	**99 (12)**	**283 (16)**	**184 (18)**	**99 (13)**
*Completed (n*,* % of telephone visits)*	*214 (88)*	*133 (92)*	*81 (82)*	*232 (82)*	*152 (83)*	*80 (81)*
*Rescheduled*	*9 (4)*	*2 (1)*	*7 (7)*	*16 (6)*	*11 (6)*	*5 (5)*
*No-show*	*13 (5)*	*6 (4)*	*7 (7)*	*28 (10)*	*17 (9)*	*11 (11)*
*Other (cancelled or walk out)*	*7 (3)*	*3 (2)*	*4 (4)*	*7 (3)*	*4 (2)*	*3 (3)*
**Video visits (n**,** % of all visits)**	**8 (< 1)**	**0 (0)**	**8 (< 1)**	**26 (1)**	**2 (< 1)**	**24 (3)**
*Completed (n*,* % of video visits)*	*5 (63)*	*0 (0)*	*5 (63)*	*15 (58)*	*1 (50)*	*14 (58)*
*Rescheduled*	*1 (12)*	*0 (0)*	*1 (12)*	*3 (12)*	*1 (50)*	*2 (8)*
*No-show*	*1 (12)*	*0 (0)*	*1 (12)*	*5 (19)*	*0 (0)*	*5 (21)*
*Other (cancelled or walk out)*	*1 (12)*	*0 (0)*	*1 (12)*	*3 (12)*	*0 (0)*	*3 (13)*

Table 3 shows associations between sociodemographic characteristics and telemedicine utilization. Women (cis and transgender) were more likely to utilize telemedicine (73% versus 50% of men, *p* < 0.05), as were those with a college education compared to those with high school or lower levels of education (69% versus 49%, *p* < 0.01). Although not statistically significant, we also found that participants identifying as White were more likely to use telemedicine compared to people of other races/ethnicities (73% versus 55% of Black/African American versus 44% Hispanic/Latino/a participants, *p* = 0.08). Additionally, there was a trend towards those with stable (versus unstable) housing being more likely to utilize telemedicine (56% versus 41%, *p* = 0.09).

**Table 3:** Sociodemographic predictors of participants engaged in care by telemedicine utilization (*n* = 257)*

	No telemedicine visits (*n* = 119)	One or more telemedicine visits (*n* = 138)	*p-value*
**Age**, (Median, IQR)	47 (38, 57)	51 (39, 60)	*p* < 0.05
**Gender**			*p* < 0.05
Cisgender male (n, % of cis male)	103 (87%)	102 (74%)	
Cisgender female (n, % of cis female)	12 (10%)	32 (23%)	
Transgender female (n, % of trans female)	2 (2%)	3 (2%)	
Gender diverse (n, % of gender diverse)	2 (2%)	1 (< 1%)	
Race/Ethnicity**			*p* = 0.08
Black/African American (n, % of Black/AA)	53 (45%)	66 (48%)	
Hispanic/Latino/a (n, % of Hispanic/Latino/a)	38 (32%)	30 (22%)	
White (n, % of White)	7 (6%)	19 (14%)	
Other (n, % of other)	20 (17%)	23 (17%)	
**Education level**			*p* < 0.01
College or more (n, % of college or more)	20 (17%)	44 (32%)	
High school or less (n, % of high school or less)	99 (83%)	94 (68%)	
**Employment status**			*p* = 0.17
Not working or on disability (n, % of not working/ on dis)	57 (48%)	78 (57%)	
Working or retired (n, % of working/ret)	62 (52%)	60 (43%)	
Housing status**			*p* = 0.09
Stable (n, % of stable)	96 (81%)	123 (89%)	
Unstable (n, % of unstable)	22 (18%)	15 (11%)	
**Preferred language**			*p* = 0.22
English (n, % of English)	103 (87%)	116 (84%)	
Spanish (n, % of Spanish)	14 (12%)	22 (16%)	
Other (n, % of other)	2 (2%)	0 (0%)	
**Clinic**			*p* = 0.53
Clinic 1 (n, % of clinic 1)	57 (48%)	72 (52%)	
Clinic 2 (n, % of clinic 2)	62 (52%)	66 (48%)	

*Only includes participants with at least one completed visit (telemedicine and/or in-person)

**Prefer not to answer, *n* = 1

### PLHIV report of clinician offer of telemedicine

At endline, approximately 70% of all participants surveyed (of *n* = 169 who responded to this question) reported that they were offered telemedicine during the intervention: 36% (*n* = 61) reported being offered both telephone and video visits; 36% (*n* = 61) reported being offered telephone only visits. The remaining individuals reported not being offered telemedicine (28%, *n* = 47). Seventeen percent (*n* = 28) reported being offered both video and telephone visits by their provider, but only experienced telephone visits. When asked why they did not have a video visit despite being offered one, the most common responses were: phone was easier to use (39%, *n* = 11); clinical provider decided to use telephone instead of video at time of visit (25%, *n* = 7); participant did not want video due to discomfort with their appearance (25%, *n* = 7); and privacy concerns with video (18%, *n* = 5). Nineteen percent of PLHIV (*n* = 32) reported being offered but declining use of telemedicine (either video or telephone). The most common reasons for declining included a desire to have a physical exam, blood draw, or office procedure at the same time as the appointment (59%, *n* = 19); preferred interpersonal dynamic with their clinician in-person (38%, *n* = 12); in-person better for absorbing information/results (13%, *n* = 4); and technological issues with phone/video (6%, *n* = 2). Table 4 summarizes telemedicine offer by type (video versus telephone).

**Table 4:** Offer of telemedicine based on client report (*n* = 172)*

	Overall (*n* = 172)	Clinic 1 (*n* = 88)	Clinic 2 (*n* = 84)
**Offered telemedicine**, n (%)			
Both video and telephone Only telephone Only video Neither (only in-person) I don’t know/remember	61 (35) 61 (35) 0 (0) 47 (27) 3 (2)	23 (26) 39 (44) 0 (0) 24 (27) 2 (2)	38 (45) 22 (26) 0 (0) 23 (27) 1 (1)
**If offered both telephone and video**,** frequency of offer**, n (%)			
Telephone and video offered equally More telephone offered than video More video offered than telephone I don’t know/remember	49 (80) 8 (13) 2 (3) 2 (3)	19 (83) 3 (13) 1 (4) 0 (0)	30 (79) 5 (13) 1 (3) 2 (5)
**If offered only telephone**,** frequency of offer**, n (%)			
Telephone and in-person equal More telephone than in-person More in-person than telephone	20 (33) 1 (2) 40 (66)	12 (31) 0 (0) 27 (69)	8 (36) 1 (5) 13 (59)

*Data included only for individuals who completed the endline survey

### Virologic outcomes

A total of 123 people had a viral load within six months of baseline and were included in the viral suppression analysis. Viral suppression rates (< 40 copies/mL) were lower at baseline among telemedicine non-users compared to users (71.0% versus 83.8%) and did not significantly change for either group over the intervention period (77.5% at endline among telemedicine non-users [*p* = 0.26] and 81.0% among telemedicine users [*p* = 0.19]) (Table 5). A sensitivity analysis using < 200 copies/mL yielded similar findings (Supplementary Table 2). There were no differences by clinic for these findings.

**Table 5:** Viral suppression (< 40 copies/mL) and telemedicine utilization over the intervention period

Quarter of intervention	Participants with in-person visits only				Participants with any telemedicine use			
	N (participants)	Undetectable viral loads (*n* = 205)	Detectable viral loads (*n* = 57)	p-value	N (participants)	Undetectable viral loads (*n* = 276)	Detectable viral loads (*n* = 72)	p-value
0 (Baseline)	59	49(71.0%)	20(29.0%)	0.26	64	57(83.8%)	11(16.2%)	0.19
1	58	73(81.1%)	17(18.9%)		82	90(81.1%)	21(18.9%)	
2	60	56(74.7%)	19(25.3%)		75	74(82.2%)	16(17.8%)	
3	57	46(78%)	13(22%)		72	55(71.4%)	22(28.6%)	
4	42	34(77.3%)	10(22.7%)		75	64(80.0%)	16(20.0%)	
5	37	31(77.5%)	9(22.5%)		52	47(81.0%)	11(19.0%)	

### Technological resources and literacy

At baseline, almost all participants (94%, *n* = 254) responded that they had a device that would allow both audio and video and had consistent network service/bandwidth needed for telephone and video visits. Participants reported very high technological literacy for all devices (TLST score, scale 0 lowest and 16 highest), with an overall median of 14 (IQR 12, 15).

### Telemedicine experience

At endline, most participants reported having privacy for telemedicine (telephone and/or video) visits over the prior year (88%, *n* = 152). Only three people who had used telemedicine visits reported that privacy was always a barrier for them (2%), and all three reported using telephone visits, but not video. Participants reported high satisfaction with telemedicine. Of the 91 respondents with telephone visit experience, 95% (*n* = 85) were satisfied compared to 100% (*n* = 10) with video visit experience. This level of satisfaction with telemedicine was similar to that for in-person visits (96% satisfied).

### Key informant interviews

Nine endline in-depth interviews were completed between September 2023 and January 2024: 6 clinicians, 2 clinic leaders, and 1 case manager. Participants had a median time working in the field of HIV of 15 years (IQR 10, 18) and a median time working at their current clinic of 8 years (IQR 4, 14). Clinicians had a median of three years of experience with providing telemedicine for HIV care, corresponding with the beginning of the stay-at-home orders of the COVID-19 pandemic.

All respondents felt that telephone visits served an important purpose within their clinics. Individuals believed that telephone telemedicine helped clients remain engaged in care and facilitated follow-up of important clinical issues that could go unaddressed if only in-person or video visits were allowed.

*I think telephone visits are serving a real purpose in care – even though I’m maybe doing that 10% of the time*,* I think that 10% is really critically important… Those are visits that would otherwise not happen*,* and those are visits that are important where people are getting lab results*,* or getting adherence counseling*,* or getting follow-up of ongoing issues*,* feeling connected to the clinic*,* feeling supported*,* and I think it would be a great disservice to not be able to deliver telephone care. (Clinician 1*,* 17 years in HIV care)*

Most respondents perceived high client interest in telephone telemedicine.

*I’ve rarely had someone refuse or decline a phone visit – it’s just those few who are like*,* ‘I want to be seen in person all that time*,*’ and that’s very few. Most are accepting. (Clinician 2*,* 19 years in HIV care)*

While telephone was felt to be almost universally acceptable for clients, some respondents felt video telemedicine was appropriate for only certain PLHIV.

[*Video is for those who] really want to do a video visit*,* they have good*,* stable technology*,* and they’re tech savvy… (Clinician 3*,* 30 years in HIV care)*

Other respondents reported that technological fluency does not always correlate with clients’ desire to use video telemedicine.

*I think my hypothesis going into the ramp up of [video] telemedicine was – my more educated*,* tech-literate patients will want it and value it and see it as a higher-quality experience – and that’s just not been the case. They don’t want it; they want telephone. (Clinician 1*,* 17 years in HIV care)*

Although clients uncommonly reported lack of privacy as a concern for use of telemedicine, including video, clinicians perceived their clients’ preferred telephone for ease of privacy.

*[The] ease*,* whether it be ease of finding privacy [or] ease of finding connectivity with phone over video*,* that tends to be the biggest reason that patients choose phone over video*…*There are barriers in terms of privacy and having a quiet space where the patient feels safe to share that information with video*,* while also not invading some of their either coworkers’ or roommates’ or partner’s or family’s privacy. A lot of my patients do tend to be in houses with multiple family groups within them; that might skew them more towards a phone visit over a video visit. (Clinician 4*,* 5 years in HIV care)*

All respondents reported that older clients and clients with more non-HIV-related comorbidities preferred in-person visits.

*Older patients just can’t conceive of doing a visit any other way [than in-person]. I think a lot of patients are judging their medical situation – they know it’s more complex and that more exams need to be done. They need to get labs done every time they come in… so they need to be here in person. (Clinician 3*,* 30 years in HIV care)*

At least one key informant perceived established patients were the population most inclined towards telemedicine visits.

*The patients that have been established in care for many*,* many*,* many years with the doctors and with our clinic are trending more towards the telehealth. (Case manager 1, 9 years in HIV care)*

Several clinicians overtly stated preferring telephone over video, with either negative attitudes about video despite the ease of the Doximity platform, or the belief that video did not provide value for most visits.

*I don’t like [Doximity]—it’s just another video format*,* as far as I’m concerned. I just don’t like video very much. (Clinician 5*,* 15 years in HIV care*)

*[Video] gives you more of the experience in person… but for the work I’m doing*,* just documenting what I need to in my record*,* the phone is basically I’d say 95%—it allows me to do what I need to do at least 95% of the time because really just communication with the patient*,* and even video*,* the exam I could get with a video camera is only going to give me part of what I would need. If I need a full exam*,* I need to see them in person. (Clinician 2*,* 19 years in HIV care)*

Many respondents raised concerns about the future of reimbursement for telephone telemedicine visits, stating this would be a disservice to PLHIV in FQHCs who have been benefiting from this modality since the start of the COVID-19 pandemic. One clinician stated that those who most struggle to remain engaged in care would be the most negatively impacted if telephone telemedicine was no longer reimbursable:

*…people who have more instability in their lives; people that have more challenges with managing their schedules; people that are struggling to balance work and family commitments; people where there is cost associated with coming to the visits; people with mental illness and substance disorders that tend to*,* in my experience*,* engage a little bit less via telemedicine but every once in a while they do and those interactions are also really important. So*,* I think the ones for whom this is helping the most are the ones that are struggling the most to engage in regular in-person care. (Clinician 1*,* 17 years in HIV care)*

This sentiment was shared by many respondents, who all hoped that telephone visits would remain available to clinic patients in the future:

*I’ve always been impressed with seeing how many patients took up the offer for a phone visit and*,* in my own personal panel*,* have seen instances where I wouldn’t have been able to complete a visit otherwise if phone wasn’t available to have the interaction with the patient. I really think this is something that has been way overdue and something that has huge benefit*,* particularly for patients who do experience a lot more of the social determinants of health in a negative way. (Clinician 4*,* 5 years in HIV care)*

## Discussion

Despite an intervention focused on improving the offer of telemedicine with an emphasis on increasing the uptake of video telemedicine at two FQHCs in Los Angeles, the proportion of people using telemedicine remained similar, but overall share of visits performed by telemedicine modestly increased. Most visits were performed by telephone and not video, despite the intervention’s emphasis on encouraging video visits. This intervention was delivered in the context of a population with good reported access to technological resources for video telemedicine, and high technological literacy. Our findings about the frequency of telemedicine utilization are counter to other reports noting a shift back to more in-person healthcare in the “post-pandemic era” [25–28], as we saw a trend suggesting more telemedicine visits over the intervention period (late 2022–2023). While at least one study has observed “telehealth fatigue” with decreasing acceptance and popularity of telemedicine since its peak use during the pandemic [29], we found universally high client satisfaction for both telephone and video telemedicine. Our findings are consistent with the literature on telemedicine for other chronic conditions (mental health, diabetes, and cardiovascular disease) in a variety of settings (FQHCs, veterans affairs, academic), which report high telephone and video satisfaction rates [4–6, 9].

We found no significant change in viral suppression rates from baseline over the intervention period for either telemedicine users or non-users. Telemedicine non-users had lower suppression at baseline, suggesting these individuals may represent a more unstable group with adherence challenges. Clinicians may have been less likely to offer telemedicine to these individuals, and/or these individuals may have declined telemedicine offers. Further studies are required to verify and further explore our findings. While not statistically significant, viral suppression rates did increase in telemedicine non-users. This may reflect an increase in care engagement for this group after the transition of COVID-19 from pandemic to endemic stages.

Several studies have raised concern that telemedicine for HIV care would lead to worse health outcomes, including a reduction in viral suppression [10, 13]. Our data suggest the use of telemedicine is not harmful with respect to viral load outcomes. A systematic review and meta-analysis of telehealth for HIV care done in clinic, hospital, and community contexts found that telehealth interventions increased adherence to treatment, reduced depressive symptoms, and improved quality of life; however, this analysis did not report on viral load outcomes [30]. Additional studies with longer follow-up in larger populations are required to confirm our findings and understand the impact of telemedicine on HIV outcomes over longer time periods.

Our findings mirror the literature showing that sociodemographics are an important determinant of telemedicine use. We found lower overall utilization among men and those with less education, with a trend toward lower use among those identifying as Black/African American and Latino/a. However, studies have shown that overall, telemedicine, particularly audio-only, does increase access for people who have traditionally struggled to remain engaged in care, including people with disabilities (specifically mobility disabilities), older adults, people who identify as Black, those whose primary language is Spanish, and those who are publicly insured [12, 31]. Further research is needed to understand the interplay between sociodemographic characteristics and telemedicine utilization by modality (telephone versus video) to ensure equitable access and achieve maximal benefit for PLHIV cared for in FQHCs.

Our results underscore the continued importance of audio-only telemedicine, as we found very high rates of visit completion for this modality (far higher than for in-person or video), and most clinicians expressed that telephone telemedicine resulted in care that would likely never have occurred without this option for follow-up. Despite our intervention’s focus on video -- including implementing an easy-to-use video platform, providing a telemedicine navigator, and scheduling regular quality improvement meetings -- clinicians remained less likely to offer video, and several clinicians retained negative views of video telemedicine. Although studies have shown that support such as navigators and education for staff helps promote video telemedicine use [32, 33], we found limited benefits for increasing the use of video. One cross-sectional study of predictors of video versus telephone telemedicine found that clinician factors (implicit bias, clinician training, and clinic infrastructure) were more important than patient factors [34]. Further research is needed to understand clinician-level barriers to telemedicine and to evaluate interventions that may facilitate the offer of telemedicine, including video, in FQHC settings.

In the context of high satisfaction and high visit completion rates, and no harm with regard to viral suppression, our data suggest that telephone telemedicine is playing an important role for PLHIV cared for in FQHCs. While reimbursement for telephone visits remains in place for Medi-Cal clients at the time of writing this manuscript, its long-term future remains uncertain. Our findings highlight the potential inequity of ending reimbursement for telephone telemedicine for PLHIV in FQHCs. The loss of this option may impact engagement in care and health outcomes of this vulnerable population.

### Limitations

Our study included only PLHIV actively engaged in care in the prior year before enrollment and therefore we cannot generalize findings to individuals returning to care or those individuals with frequent and/or prolonged treatment interruptions. It is possible these individuals could also benefit from telemedicine, and future studies should explore telemedicine use in this population. In addition, the short duration of follow-up and small sample size that had viral load data near the time of our baseline timepoint may have limited our ability to find statistically significant changes in viral suppression rates as a result of this intervention. Non-users of telemedicine had lower baseline viral suppression rates compared to users, suggesting differences in the two groups, which should be explored through larger studies with longer follow-up of viral suppression rates. Lastly, we used self-report to assess technological literacy using a novel scale (with limited data on validity in different contexts) [23], and participants may have reported higher literacy due to social desirability bias [35], so we cannot exclude that low rates of video use were the result of inexperience or other barriers to using this technology.

## Conclusions

Telephone telemedicine is an important component of health care for PLHIV in care at FQHCs in south Los Angeles and remains so well beyond the transition of COVID-19 from pandemic to endemic stages. Both clinicians and PLHIV in our study reported strong satisfaction with telemedicine. Low use of video telemedicine despite our intervention may be due, in part, to clinician factors affecting offer of video visits, and is an important area for future research, as is the study of long-term viral suppression rates among larger populations of telemedicine users and non-users in FQHC settings. The possible end of reimbursement for telephone telemedicine due to federal policies could widen disparities for PLHIV.

## Supplementary Information

Below is the link to the electronic supplementary material.

Supplementary Material 1

Supplementary Material 2

## Data Availability

The datasets used and/or analyzed during the current study are available from the corresponding author on reasonable request.

## Abbreviations

ART: Antiretroviral Therapy
CFIR: Consolidated Framework for Implementation Research
COVID-19: Coronavirus disease-19
FQHC: Federally Qualified Health Center
PLHIV: Person(s) living with HIV
RE-AIM: Reach, Effectiveness, Adoption, Implementation, and Maintenance
TLST: Telehealth Literacy Screening Tool
