# Supplementary material for: High usage of telephone telemedicine among people living with HIV at two federally qualified health centers in Los Angeles
Source: BMC Health Serv Res. 2026 May 14;26:954. doi: 10.1186/s12913-026-14707-8 (PMC13361741; doi:10.1186/s12913-026-14707-8)
Supplement: Supplementary file 1 — Supplementary Material 1 [file 12913_2026_14707_MOESM1_ESM.docx]

Clinician In-Depth Interview Guide (Endline)

*Before starting this section confirm oral consent has been given, that the participant has verbalized “yes” they will participate and “yes” they consent to recording the interview. Remind the participant that they should not say their own name or any other information that could inadvertently identify them during the interview. The interviewer should not use the participant’s name during the interview to keep the data anonymized. It is important for the participant to not give any identifying information about themselves, patients, or other clinicians/clinic staff. Instead of saying their names, they can say “my colleagues”, “my patient”, or “the case managers”, for example.*

**START RECORDING**

First, I am going to ask a few questions about your background and your role in caring for patients living with HIV at your clinic.

**SOCIO-DEMOGRAPHICS**

1. What is your role in clinic (doctor, RN, other & describe)?
2. For how many years have you worked in HIV care?
3. For how many years have you worked at this clinic?
4. For how many years have you been providing telemedicine care (defined as a health care visit done via phone or video) for people with HIV?
5. Do you have experience providing telemedicine for other types of care (primary care, mental health care, other)? If yes, how many years of experience do you have doing this?

Now we will move on to the interview questions. Please feel free to ask for any clarification if you do not understand a question and take as much time as you need to think about your responses.

**INTERVIEW QUESTIONS**

1. Can you share more about your experience with telemedicine for HIV care over the last year:
   - 1. About what proportion of your patient appointment visits are currently in-person, versus by telephone, versus by video (prompt: estimate for each: one-third, half, etc)? *ask as a survey question
     2. How often do you offer your patients a telemedicine appointment for a future visit (every time, most of the time, half the time, less than half the time, never)?
        1. Of these, how often do you offer these as telephone appointment visits when appropriate (based on your own clinical expertise) for a future visit (every time, most of the time, half the time, less than half the time, never)? *ask as a survey question
        2. How often do you offer your patients video visits when appropriate (based on your own clinical expertise) for a future visit (every time, most of the time, half the time, less than half the time, never)? *ask as a survey question
     3. What are the reasons you may offer one type of visit and not another for a specific visit?
     4. Can you describe the language that you use to introduce and offer telephone visits to your patients? For video visits?
        1. If you offer video, do you show the patient how to use Doximity (if they do not know/have not used it before)? Why or why not?
     5. What barriers do you experience to offering telephone? What about video visits? *(PROMPT: clinician-level like personal biases and lack of technological knowledge, clinic-level like workflows and scheduling processes, patient-level issues like access to technological resources and privacy)*
2. How has Doximity changed your perspective on video telemedicine? Or, if not, why not?
   - 1. With the addition of Doximity, how has this changed your practice of offering and using video telemedicine? *(What benefits/challenges did you experience)* Or, if not, why not?
3. *If video experience with Doximity,* how was it? (Challenges and benefits, experience with Doximity compared to previous platforms)
   - 1. Do you feel that video visits are better/worse for your ability to manage your patients’ health versus telephone visits? What about compared to in-person visits? Why?
        1. What do you see as the appropriate use of video telemedicine, meaning what health care services or for what types of clients are they best suited for?
     2. What is your experience of uptake of video appointments (ie do patients agree to have video appointments when offered? Is this different from the uptake of telephone visits?)
        1. If they decline video, what are the reasons provided?
        2. Does the level of uptake of video visits seem to differ across different types of patients (by age, stable & suppressed, education level, language)
4. What characteristics make someone a good candidate for completing a scheduled video visit? What about for a telephone visit? (*PROBE: more educated, more adherent to care, stably housed, English speaking, etc*)
   - 1. Which type of visit is missed the most by patients (video, telephone, or in-person visits)? How often is this type of visit missed (*ask for a percent estimate*)?
     2. Which type of visit is missed the least (video, telephone, or in-person visits)? How often is this type of visit missed (*ask for a percent estimate*)?
     3. Is there a trend of patients liking one type of telemedicine visit more than the other (video or telephone)? If so, are there certain types of patients who like certain types of visits, and do you tailor your offer to certain patient characteristics?

**[Transition Language]:** We are more than halfway through the interview now. The next questions will focus on the process and logistics of telemedicine care for HIV*.*

1. Do you feel adequately trained to provide HIV care via telemedicine, specifically video visits using Doximity?

IF YES: where and when did you receive this training? What skills do you think are most important?

IF NO: What skills or knowledge do you think you are missing? What would be your preferred way to obtain these skills?

- - 1. What training still needs to be done to address what you need to continue to do telephone and video visits with your patients?

1. What is the clinic-level workflow for scheduling video visits and does this differ from your personal workflow?
   1. How efficient and smooth is the current appointment workflow for telemedicine working at your clinic (eg not your personal workflow if you have adjusted it)?
      1. Do you have any suggestions to improve the current workflow for telemedicine?
2. How do you think patients should be informed that telephone and video visits are available to them (i.e., advertising with flyers or text messages, offered by front desk staff, etc.)?
   - 1. What are ways that it can be offered equitably so that all patients have the opportunity to participate if they desire?
3. Is there anything the leadership of this clinic could do to help support the delivery of telemedicine visits for patients?
   - 1. How about for video visits specifically? For telephone visits specifically?
        1. If patients wanted more video visits, what would you need in order to be able to offer this as appropriate? *(PROMPT: dedicated telemedicine time, dual screen, better computer, etc.)*
     2. Have there been things the clinic leadership has tried over the last year in support of telemedicine that worked well? That didn’t work? Did this vary for video versus telephone? (If someone shares that leadership tried something and it failed, probe for what failed by saying “What was clinical leadership trying to accomplish? Why do you think it wasn’t successful?)
4. There is a possibility that reimbursement for telemedicine will change in the future such that only video visits will be reimbursable.
   - 1. If mostly using telephone, do you think you would start using more video visits if telephone visits are no longer reimbursed?
     2. How would it impact your patients if telephone visits are no longer reimbursed and go away? *(PROMPT: for engagement and retention, for viral load suppression, for comorbidity management, for mental health, etc.)*
5. Is there anything else you would like to share about your experience or thoughts about telemedicine for HIV care in the past year that we did not cover in this interview?

**END RECORDING**

Non-Clinician In-Depth Interview Guide (Endline)

*Before starting this section confirm oral consent has been given, that the participant has verbalized “yes” they will participate and “yes” they consent to recording the interview. Remind the participant that they should not say their own name or any other information that could inadvertently identify them during the interview. The interviewer should not use the participant’s name during the interview to keep the data anonymized. It is important for the participant to not give any identifying information about themselves, patients, or other clinicians/clinic staff. Instead of saying their names, they can say “my colleagues”, “my patient”, or “the case managers”, for example.*

**START RECORDING**

First, I am going to ask a few questions about your background and your role in supporting patients living with HIV at your clinic.

**SOCIO-DEMOGRAPHICS**

1. What is your role in clinic (case manager, administrator, other & describe)?
2. For how many years have you worked in HIV care?
3. For how many years have you worked at this clinic?

Now we will move on to the interview questions. Please feel free to ask for any clarification if you do not understand a question and take as much time as you need to think about your responses.

**INTERVIEW QUESTIONS**

1. Can you share more about your role and how you have personally been involved with telemedicine for HIV care over the last year:
   - 1. How do you specifically support telemedicine (schedule appointments; participate in visits; educate or support patients)?
     2. About what proportion of patient appointment visits are currently in-person, versus telephone, versus by video (one-third, half, etc.)? *ask as a survey question
     3. How often do you discuss telemedicine with patients (every day, every week, every month, less often than every month)? *ask as a survey question
        1. What do these discussions entail?
     4. How often do you discuss telemedicine with colleagues (every day, every week, every month, less often than every month)? *ask as a survey question
        1. What do these discussions entail?
2. How has the addition of Doximity changed your perspective on telephone and video telemedicine? Or, if not, why not?
   - 1. With the addition of Doximity, how has this changed the practice of telephone and video telemedicine at your clinic? How? *(What benefits/challenges did you/your clinic experience)* Or, if not, why not?
3. What is your perception of the video visit experience at your clinic? (Challenges and benefits for patients and clinicians, experience with Doximity compared to previous platforms)
   - 1. What is your sense of the uptake of video appointments at your clinic (i.e., do patients agree to have video appointments when offered? Is this different from the uptake of telephone visits?)
4. What characteristics make someone a good candidate for completing a scheduled video visit? And for a telephone visit? (*PROBE: more educated, more adherent to care, stably housed, English speaking, etc.*)
5. Which type of visit is missed the most by patients (video, telephone, or in-person visits)? How often is this type of visit missed (*ask for a percent estimate*)?
6. Which type of visit is missed the least (video, telephone, or in-person visits)? How often is this type of visit missed (*ask for a percent estimate*)?
7. Is there a trend of patients liking one type of visit more than the other? If so, are there certain types of patients who like certain types of visits (age, language spoken, newer vs more established patient, literacy level)?

**[Transition Language]:** We are more than halfway through the interview now. The last few questions will focus on the process and logistics of telemedicine care for HIV*.*

1. Do you feel you received adequate information regarding telemedicine, specifically video visits using Doximity, to support it generally in the role that you play?

IF YES: where and when did you receive this information? What additional information would you like?

IF NO: What information do you think is missing? What would be your preferred way to obtain this information?

- 1. What training still needs to be done to address what you need in your role as it relates to improving telemedicine at your clinic?

1. What is the clinic-level workflow for scheduling video visits and does this differ from your personal workflow?
   1. How efficient and smooth is the current appointment workflow for telemedicine working at your clinic (vs your personal workflow, if different)?
   2. Do you have any suggestions to improve the current workflow for telemedicine?
2. How do you think patients should be informed that telephone and video visits are available to them (i.e., flyers, text messages, conversations with staff, etc.)?
   1. What are ways that it can be offered equitably so that all patients have the opportunity to participate if they desire?
3. Is there anything the leadership of this clinic could do to help support the delivery of telemedicine visits for patients?
   - 1. How about for video visits specifically? For telephone visits specifically?
     2. Are there any clinic-level barriers that make delivery of telemedicine visits challenging? Any specific differences in barriers between video and telephone? *(PROMPT: workflows, scheduling processes)*
     3. Have there been things the clinic leadership has tried over the last year in support of telemedicine that worked well? That didn’t work? Did this vary for video versus telephone? (If someone shares that leadership tried something and it failed, probe for what failed by saying “What was clinical leadership trying to accomplish? Why do you think it wasn’t successful?)
4. There is a possibility that reimbursement for telemedicine will change in the future such that only video visits will be reimbursable.
   - 1. If your clinic is mostly using telephone, do you think clinicians would start using more video visits if telephone visits are no longer reimbursed?
     2. How would it impact your patients if telephone visits are no longer reimbursed and go away? *(PROMPT: for engagement and retention, for viral load suppression, for comorbidity management, for mental health, etc.)*
5. Is there anything else you would like to share about your experience or thoughts about telemedicine for HIV care in the past year that we did not cover in this interview?

**END RECORDING**
