# Supplementary material for: High usage of telephone telemedicine among people living with HIV at two federally qualified health centers in Los Angeles
Source: BMC Health Serv Res. 2026 May 14;26:954. doi: 10.1186/s12913-026-14707-8 (PMC13361741; doi:10.1186/s12913-026-14707-8)
Supplement: Supplementary file 2 — Supplementary Material 2 [file 12913_2026_14707_MOESM2_ESM.docx]

**Client Endline Survey**

| SECTION 1. SOCIODEMOGRAPHIC | |
| --- | --- |
| 1. PID | ### |
| 1. What gender do you identify as? | - Female - Male - Transgender female - Transgender male - Gender nonconforming - Other (please describe): __________ - Prefer not to answer |
| 1. What is your race/ethnicity? Select all that apply. | - Black/African-American - Hispanic or Latino/a - Asian - Native American - White/Caucasian - Other (please describe): __________ - Prefer not to answer |
| 1. What language do you prefer to use when communicating with your clinician? 2. On a scale of 1-5, how comfortable are you with reading in this language? | - English - Spanish - Other: __________   1 2 3 4 5  *Not Totally at all comfortable comfortable* |
| 1. If English is not your preferred language, on a scale of 1-5, how comfortable are you with speaking and understanding English? 2. On a scale of 1-5, how comfortable are you with reading English? | 1 2 3 4 5  *Not Totally at all comfortable comfortable*  1 2 3 4 5  *Not Totally at all comfortable comfortable* |
| 1. What sexual orientation do you most identify with? | - Heterosexual/straight - Gay - Lesbian - Queer - Bisexual - Pansexual - Asexual - Other: __________ - Prefer not to answer |
| 1. What is the highest level of school you have completed? | - None - Some school but did not complete high school/GED - High school/GED - College/university - Graduate studies - Do not know - Prefer not to answer |
| 1. What was the year of your HIV diagnosis?   [okay to approximate if not certain] | - YYYY - Do not know - Prefer not to answer |
| 1. When did you first start antiretroviral therapy (ART)? | - MM/YYYY - Do not know - Prefer not to answer |

| SECTION 2. TELEMEDICINE HISTORY | |
| --- | --- |
| 1. How is this survey being completed? | - In-person - Telephone - Zoom |
| 1. Over the last year, have you had any telephone/audio or video telemedicine visits for your HIV care (defined as a scheduled appointment via telephone or video to provide HIV-related primary care)? | - Yes, telephone/audio only - Yes, video only - Yes, both telephone/audio and video - No - I don’t know/remember - Prefer not to answer |
| 1. What kind of telemedicine visits have you been offered (from your doctor or other clinic staff)?   If telephone/audio,   1. How often were you offered telephone/audio visits in the last year?   If video,   1. How often were you offered telephone/audio visits in the last year?   If you were offered both and have only used telephone visits,   1. Why did you choose telephone over video visits? (Select all that apply)   If you were offered both and have only used video visits,   1. Why did you choose video over telephone? (Select all that apply)   If you were offered both and have used both,   1. What makes you pick one option over the other for a visit? (Select all that apply)   If you were offered telemedicine and have not used any telephone or video visits,   1. Why did you choose in person over telephone or video visits? (Select all that apply)   If you were offered only telephone visits,   1. Would you have wanted to be offered video visits also?   If you were offered only video visits,   1. Would you have wanted to be offered telephone visits also?   If you were offered only in-person visits,   1. Would you have wanted to be offered telephone and/or visits also? | - Telephone/audio only - Video only - Both telephone/audio and video - Neither - I don’t know/remember - Prefer not to answer - 1 time - 2 times - 3 times - 4 or more times - I don’t know/remember - 1 time - 2 times - 3 times - 4 or more times - I don’t know/remember - Environmental privacy concerns (e.g. don’t have a private room) - Digital privacy concerns (e.g. don’t trust personal information on the internet) - Lack of reliable access to internet connection - Don’t know how to use my device for video calls/visits - Lack of a phone or tablet/computer with a functioning camera - Phone is more convenient - Phone is more simple to use - Prefer not to be on camera with the doctor able to see their environment - Prefer not to be on camera with the doctor able to see them (uncomfortable with the way they look on camera) - Accessibility issues for certain disabilities, like visual impairments - Simpler to receive language interpretation over phone - Can better absorb information via verbal conversations versus visual conversations - Had a previous bad experience with video - Other: ___________ - Allows for doctor to do a visual assessment of any physical issues - Allows for doctor to see medication bottles for medication management - Better for interpersonal dynamic and relationship with doctor - Doctor can show results or other helpful images - Prefer to be on camera so that doctor can see them/their environment - Simpler to receive language interpretation over video - Can better absorb information via visual conversations versus verbal conversations - Had a previous bad experience with telephone - Other: ___________ - Have environmental privacy only sometimes - Have digital privacy concerns only sometimes - Have reliable Internet only sometimes - Has a family member or friend who is only available to help them sometimes - Have something to physically show the doctor only sometimes - Have a device with a camera only sometimes - Feel like being on camera only sometimes - Other: ___________ - Allows for doctor to do physical assessment including blood pressure and other office procedures (glucose check) - Can get blood drawn and/or do labs at same time - Can pick up medications at same time - Can meet with case manager/counselor at same time - Can receive other services at same time (i.e. insurance enrollment, TAP card, etc.) - Better for interpersonal dynamic and relationship with doctor - Better for urgent issues - Doctor can show results or other helpful images - Easier with language barriers due to English not being dominant language - Can better absorb information in person versus virtually - Had a previous bad experience with telephone or video - Other: ___________ - Yes - No - I don’t know - Yes - No - I don’t know - Yes, telephone only - Yes, video only - Yes, both telephone and video - No - I don’t know |
| IF NO EXPERIENCE WITH TELEMEDICINE, SKIP QUESTIONS 24-39  On a scale from 1 to 4, with 1 being Strongly disagree and 4 being Strongly agree, how do you feel about the following statements? Below telemedicine includes both telephone and video visit types.   1. Telemedicine for HIV care is more convenient than in-person visits. 2. I am more likely to miss an HIV care telemedicine appointment than an in-person appointment. 3. I am more likely to be late to an HIV care telemedicine appointment than an in-person appointment. 4. Telemedicine for my HIV care saves me time compared to in-person visits. 5. Telemedicine for my HIV care saves me money compared to in-person visits.   Note: participants should consider the total money for their visit, such as the money they spend on traveling to the clinic or the money they lose from taking time off from work.   1. I trust my doctor to offer me a telemedicine visit when it is appropriate for my HIV care. 2. I trust my doctor to give me the choice between telephone and video visits when it is appropriate for my HIV care. 3. It is easy to maintain the kind of relationship I like best with my doctor during telemedicine HIV care versus in-person. 4. I feel comfortable being on a video telemedicine call for HIV care with my doctor where they can see me and I can see them. *SKIP IF HAVEN’T USED VIDEO 5. I feel more comfortable talking with my HIV care doctor about sensitive topics over a phone/audio call than in-person. *SKIP IF HAVEN’T USED TELEPHONE 6. I feel more comfortable talking with my HIV care doctor about sensitive topics over a video call than in-person. *SKIP IF HAVEN’T USED VIDEO 7. I feel more comfortable talking with my HIV care doctor about sensitive topics over video call than phone/audio call. *SKIP IF HAVEN’T USED TELEPHONE AND VIDEO 8. I feel satisfied with the quality of care I’ve received on my HIV phone/audio visits. *SKIP IF HAVEN’T USED TELEPHONE 9. I feel satisfied with the quality of care I’ve received on my HIV video visits. *SKIP IF HAVEN’T USED VIDEO | - 1 – Strongly disagree - 2 – Disagree - 3 – Agree - 4 – Strongly agree - No preference / I do not know - 1 – Strongly disagree - 2 – Disagree - 3 – Agree - 4 – Strongly agree - No preference / I do not know - 1 – Strongly disagree - 2 – Disagree - 3 – Agree - 4 – Strongly agree - No preference / I do not know - 1 – Strongly disagree - 2 – Disagree - 3 – Agree - 4 – Strongly agree - No preference / I do not know - 1 – Strongly disagree - 2 – Disagree - 3 – Agree - 4 – Strongly agree - No preference / I do not know - 1 – Strongly disagree - 2 – Disagree - 3 – Agree - 4 – Strongly agree - No preference / I do not know - 1 – Strongly disagree - 2 – Disagree - 3 – Agree - 4 – Strongly agree - No preference / I do not know - 1 – Strongly disagree - 2 – Disagree - 3 – Agree - 4 – Strongly agree - No preference / I do not know - 1 – Strongly disagree - 2 – Disagree - 3 – Agree - 4 – Strongly agree - No preference / I do not know - 1 – Strongly disagree - 2 – Disagree - 3 – Agree - 4 – Strongly agree - No preference / I do not know - 1 – Strongly disagree - 2 – Disagree - 3 – Agree - 4 – Strongly agree - No preference / I do not know - 1 – Strongly disagree - 2 – Disagree - 3 – Agree - 4 – Strongly agree - No preference / I do not know - 1 – Strongly disagree - 2 – Disagree - 3 – Agree - 4 – Strongly agree - No preference / I do not know - 1 – Strongly disagree - 2 – Disagree - 3 – Agree - 4 – Strongly agree - No preference / I do not know |
| 1. When you were newly diagnosed, how soon after would you have felt comfortable having an HIV visit via telemedicine instead of in-person? | - 3 months - 6 months - 1 year - More than 1 year - Never - No preference / I do not know |
| 1. In the future, what is your preferred mix of HIV care appointment types? | - All in-person - All telemedicine (either video or telephone) - Some in-person and some telemedicine - Not sure - No preference |
| 1. If yes to at least some telemedicine, how would you like to receive your telemedicine care? | - All telephone/audio - All video - Some telephone/audio and some video - Not sure - No preference |
| 1. In the last year, have you had telemedicine visits for any other kind of non-HIV health care? 2. If yes, did you receive this non-HIV care via telephone/audio call or video call? | - No - Yes: - Mental health care - Case management - Acute care (sinus infection, cold, flu, etc.) - COVID-related care - Specialty care (cardiology, gynecology, nephrology, etc) - Other: __________ - Do not know/remember - Prefer not to answer - Telephone/audio only - Video only - Both - Do not know/don’t remember - Prefer not to answer |
| If yes: On a scale from 1 to 4, with 1 being Strongly disagree and 4 being Strongly agree, how do you feel about the following statement?   1. I feel (or would feel) more comfortable having telemedicine visits for health issues that are not HIV-specific (like HIV treatment, adherence, STIs, etc.). | - 1 – Strongly disagree - 2 – Disagree - 3 – Agree - 4 – Strongly agree - No preference / I do not know |

| SECTION 3. ACCESS (TECH RESOURCES, HOUSING, EMPLOYMENT) | |
| --- | --- |
| 1. Do you own a phone that currently can make and receive calls? 2. If yes, what kind of phone do you have? 3. Do you use video calls on your phone to speak with family or friends, for work, or for any other purpose? *SKIP IF LANDLINE OR CELL PHONE THAT ISN’T CAPABLE OF VIDEO CALLS | - Yes - No - Do not know - Prefer not to answer - Landline - Cell phone - Smartphone (not Obamaphone) - Smartphone (Obamaphone) - Other: __________ - Do not know - Prefer not to answer - Yes - No - Prefer not to answer |
| 1. Has this or any other phone been available to you consistently over the past 3 months? | - Yes - No - Prefer not to answer |
| 1. Do you currently own a tablet or a laptop/computer? 2. If yes, do you use your tablet or laptop/computer for video calls with family of friends, for work, or for any other purpose? 3. If no, can you borrow a tablet or laptop/computer (from a friend or family member) for an HIV telemedicine visit?   (note: this is about access to the device not privacy for use of the device) | - Yes - No - Prefer not to answer - Yes - No - Prefer not to answer - Yes, consistent access (always) - Yes, inconsistent access (sometimes) - No access (never) - Prefer not to answer |
| 1. Do you currently have access to a reliable Wi-Fi or mobile data connection to use for telemedicine visits? | - Yes, always - Yes, sometimes - No, never - Prefer not to answer |
| *For the following series of questions, certain responses are assigned numeric values, which will be added at the end to give a final telehealth literacy value (add calculate function in SurveyCTO; minimum:0 and maximum:16).   1. Do you have internet at your home?   If answered yes or sometimes to having internet:   1. What devices do you use to go on the internet? (Select all that apply)   If you use a smartphone or tablet,   1. Who provides your internet access? 2. Does someone else help you use your smartphone or tablet?   *Please answer the following questions to the best of your ability. The questions relate to your baseline use and exclude the help of another individual.*  If you use a smartphone or tablet,   1. Are you comfortable with typing words into this device, such as text messaging?   If you use a smartphone or tablet,   1. Are you comfortable with opening and exiting different windows, e.g. entering your email or messages and then going back to your home page?   If you use a smartphone or tablet,   1. Are you comfortable with scrolling down a page?   If you use a smartphone or tablet,   1. Are you comfortable with selecting buttons or clicking boxes, such as “yes” or “no”?   If you use a smartphone or tablet,   1. Are you comfortable using your patient portal?   If you use a smartphone or tablet,   1. Are you comfortable with opening your email that is connected to your patient portal account?   If you use a smartphone or tablet,   1. How often do you go to your patient portal?   If you use a smartphone or tablet,   1. How often do you open your email associated with your patient portal account? | - Yes - Sometimes: ________ (please explain) - No - I don’t know - Computer - Smartphone - Tablet - A cellular data plan (through a phone company like Verizon or T-Mobile) - Wi-Fi (through a cable company like Cox) - Yes: _________ (please explain) - No - Yes (2) - Sometimes (1) - No or unsure (0) - Yes (2) - Sometimes (1) - No or unsure (0) - Yes (2) - Sometimes (1) - No or unsure (0) - Yes (2) - Sometimes (1) - No or unsure (0) - Yes (2) - Sometimes (1) - No or unsure (0) - Yes (2) - Sometimes (1) - No or unsure (0) - Frequently (2) - Sometimes (1) - Never or unsure (0) - Frequently (2) - Sometimes (1) - Never or unsure (0) |
| 1. What is your employment status? | - Working part-time - Working full-time - Retired - On disability - Not working - Prefer not to answer |
| 1. Where have you regularly slept in the past three months (pick the option that best represents the past three months)? | - Housing you own - Housing you rent - Housing you share with friends, family or acquaintances but do not pay for - Shelter, safe haven, or transitional housing - Car, in a tent, on the street, or under a bridge - Other: __________ - Prefer not to answer |
| If currently in stable housing over the past three months [skip if living in transitional or unhoused environment],   1. What is the number of individuals in your household currently who are less than 18 years old? 2. What is the number of individuals in your household currently who are 18 years or older? | ###   - Do not know - Prefer not to answer   ###   - Do not know - Prefer not to answer |
| 1. Are you worried that in the next three months you may not have stable housing that you own, rent, or stay in as part of a household? | - Yes, very worried - Somewhat worried - No, not worried at all - Prefer not to answer |
| 1. Do you have access to privacy for phone/audio calls about your HIV care in your current housing space? 2. If sometimes or no, have you found this lack of consistent privacy a barrier to having your HIV care over the phone/audio? *SKIP IF HAVEN’T HAD TELEPHONE VISITS | - Yes, always - Sometimes - No, never - Prefer not to answer - Yes, always - Sometimes - No, never - Prefer not to answer |
| 1. Do you have access to privacy for video calls about your HIV care in your current housing space? 2. If sometimes or no, have you found this lack of consistent privacy a barrier to having your HIV care over video? *SKIP IF HAVEN’T HAD VIDEO VISITS | - Yes, always - Sometimes - No, never - Prefer not to answer - Yes, always - Sometimes - No, never - Prefer not to answer |

| SECTION 4. CLINIC COSTS | |
| --- | --- |
| 1. What is the most common mode of transportation you use to get to the clinic? 2. How much time do you spend traveling one-way to the clinic on your primary mode of transportation? Round to the nearest 15-minutes. Think of the most recent few times you traveled to the clinic when you answer this question to come up with your estimate. | - Public transportation - Personal vehicle that I drive myself, like a car or motorcycle - Personal vehicle that a friend/family member drives - Ridesharing apps, like Uber or Lyft - Walking - Bicycle - Other (i.e., Access rides, ambulette services, split between modes): __________ - Do not know - Prefer not to answer   HH:MM   - Do not know - Prefer not to answer |
| 1. Do you pay anything for transportation to and from the clinic (based on the most common type of transport above)? | - Yes - No - Do not know - Prefer not to answer |
| 1. If yes, how much do you spend one-way to the clinic on your primary mode of transportation per visit? Round to the nearest dollar and think about the last time you used this most common type of transport. | $$$   - Do not know - Prefer not to answer |
| 1. Do you usually take time off from work in order to attend your HIV appointments? | - Yes - No - Sometimes - Prefer not to answer |
| 1. If yes, is this time paid or unpaid? 2. If unpaid or it varies, what is the average lost wage for the time you take off from work for an HIV appointment? Round to the nearest dollar. Think of the most recent few times you missed work when you answer this question to come up with your estimate. | - Paid - Unpaid - It varies - Do not know - Prefer not to answer   $$$   - Do not know - Prefer not to answer |
| 1. Are there any additional costs or things you spend money on to be able to attend your HIV visits at the clinic, such as childcare or an appointment companion? 2. If yes, what is the thing you spend money on? 3. What is the average additional cost for this/these for one HIV appointment (round to nearest dollar)? | - Yes - No - Do not know - Prefer not to answer - Childcare costs - Appointment companion - Other: __________ - Do not know - Prefer not to answer   $$$   - Do not know - Prefer not to answer |
| 1. Thinking about the last few times you went to the clinic in-person, how much time did you spend there, from the moment you walked in the clinic door to the moment you walked out? Round to the nearest half hour. | HH:MM   - Do not know/Don’t remember - Prefer not to answer |
| 1. How much do you pay for your phone each month, on average? This should include the cost of the actual phone if on a payment plan plus the cost of data together. 2. If $$$ > $0, how much of what you pay is just for your data plan? 3. Calculate how much of what the participant pays is just for their phone payment = Q81-Q82. 4. How do you pay for your mobile data? 5. On a scale from 1 to 10, with 1 being Not at all difficult and 10 being Extremely difficult, how difficult is it to financially maintain your phone? 6. On a scale from 1 to 10, with 1 being Not at all difficult and 10 being Extremely difficult, how difficult is it to financially maintain your mobile data? | $$$   - Do not know - Prefer not to answer   $$$   - Do not know - Prefer not to answer   $$$   - Do not know - Prefer not to answer - Monthly - Pay as you go - Other: __________ - Do not know - Prefer not to answer   1 2 3 4 5 6 7 8 9 10  *Not Totally*  *at all able able to to*   - Not applicable - Prefer not to answer   1 2 3 4 5 6 7 8 9 10 *Not Totally*  *at all able able to to*   - Not applicable - Prefer not to answer |
| 1. Do you pay for WiFi? 2. If yes or sometimes, how do you pay for your WiFi? 3. How much do you pay for your WiFi each month, on average? Round to the nearest dollar and think about the last few months to come up with your estimate. 4. On a scale from 1 to 10, with 1 being Not at all difficult and 10 being Extremely difficult, how difficult is it to financially maintain your WiFi? | - Yes - Sometimes - No - Do not know - Prefer not to answer - Monthly - Pay as you go - Other: __________ - Do not know - Prefer not to answer   $$$   - Do not know - Prefer not to answer   1 2 3 4 5 6 7 8 9 10 *Not Totally*  *at all able able to to*   - Not applicable - Prefer not to answer |
